# Infant Cries Rattle Adult Cognition

**DOI:** 10.1371/journal.pone.0154283

**Published:** 2016-05-18

**Authors:** Joanna Dudek, Ahmed Faress, Marc H. Bornstein, David W. Haley

**Affiliations:** 1 Department of Psychology, University of Toronto, Toronto, ON, Canada; 2 Eunice Kennedy Shriver National Institute of Child Health and Human Development, Bethesda, Maryland, United States of America; Goldsmiths, University of London, UK, UNITED KINGDOM

## Abstract

The attention-grabbing quality of the infant cry is well recognized, but how the emotional valence of infant vocal signals affects adult cognition and cortical activity has heretofore been unknown. We examined the effects of two contrasting infant vocalizations (cries vs. laughs) on adult performance on a Stroop task using a cross-modal distraction paradigm in which infant distractors were vocal and targets were visual. Infant vocalizations were presented before (Experiment 1) or during each Stroop trial (Experiment 2). To evaluate the influence of infant vocalizations on cognitive control, neural responses to the Stroop task were obtained by measuring electroencephalography (EEG) and event-related potentials (ERPs) in Experiment 1. Based on the previously demonstrated existence of negative arousal bias, we hypothesized that cry vocalizations would be more distracting and invoke greater conflict processing than laugh vocalizations. Similarly, we expected participants to have greater difficulty shifting attention from the vocal distractors to the target task after hearing cries vs. after hearing laughs. Behavioral results from both experiments showed a cry interference effect, in which task performance was slower with cry than with laugh distractors. Electrophysiology data further revealed that cries more than laughs reduced attention to the task (smaller P200) and increased conflict processing (larger N450), albeit differently for incongruent and congruent trials. Results from a correlation analysis showed that the amplitudes of P200 and N450 were inversely related, suggesting a reciprocal relationship between attention and conflict processing. The findings suggest that cognitive control processes contribute to an attention bias to infant signals, which is modulated in part by the valence of the infant vocalization and the demands of the cognitive task. The findings thus support the notion that infant cries elicit a negative arousal bias that is distracting; they also identify, for the first time, the neural dynamics underlying the unique influence that infant cries and laughs have on cognitive control.

## Introduction

As Charles Darwin [1] and many others since have observed, infants grab our attention. The highly salient nature of infant cues may preferentially recruit adult attention and enhance processing of infant stimuli, increasing infant survival [2]. Although the cognitive implications of this standard evolutionary view have not heretofore been systematically questioned, the salience of infant signals and their activation of a caregiving system may disrupt the adult’s capacity to process information and make important decisions, potentially affecting the care of a child. Thus, while there has been much interest in adult attention biases to infants, how infant signals influence the cognitive control processes needed to coordinate thoughts and actions has remained unclear.

Relevant to the question of how the emotional context of infant signals influences adult cognitive control, a growing body of research has examined the effects of infant stimuli on the capture of attention. Consistent with the proposal that biologically relevant stimuli demand increased allocation of attention [3, 4], infants and threats have been shown to similarly capture visual attention by activating an orienting response [5]. In terms of relative biological relevance, infant emotional faces capture more attention (producing slower performance on a target task) than adult emotional faces [6, 7], consistent with the idea that attachment-like and/or infant stimuli are more salient than adult social stimuli. In terms of valence, there is mixed evidence that negative vs. positive infant facial expressions differentially modulate attention capture [7, 8]. In contrast to the null findings reported by Thompson-Booth et al. [7], who utilized a visual attention capture paradigm, Pearson et al. [8] showed that performance on a Go/No-Go task was 25 milliseconds slower after viewing a photograph of a distressed vs. non-distressed infant in the background.

Although much of the literature has focused on the infant face, the infant voice constitutes another powerful signal that potentially modulates attention and information processing [9]. The acoustical features of the infant cry are thought to elicit alertness and distress [10]. For example, cry pitch is noted to be an influential factor shaping caregiver perceptions and responses [11], with higher-frequency cries perceived as more aversive and distressing [12]. Brain imaging studies have shown that infant vocalizations activate cortical regions involved in cognitive control and attention (e.g., the prefrontal cortex [PFC], anterior cingulate cortex [ACC], and orbitofrontal cortex), motivation and reward (e.g., the medial preoptic area [MPOA], striatum, nucleus accumbens, and substantia nigra), and affect/emotion (e.g., the amygdalae, hypothalamus, and insula) [13–16]. Electrocortical responses to infant vocalizations provide further insight into how infant vocalizations affect attentional systems [17, 18]. For example, Purhonen et al. [18] found larger amplitudes in response to infant cries than to control sounds in the N100 event-related potential (ERP), an index of the orienting response. Furthermore, physiological responses to infant cries produce increases in autonomic arousal in adults [19, 20], which may motivate sensitive or responsive parenting [21].

Infant cry vocalizations have been shown to negatively affect cognitive control by reducing concentration in parents [22, 23] and in non-parents [23]. For example, Morsbach et al. [22] asked mothers to solve arithmetic problems while hearing a healthy infant cry, a brain-damaged infant cry, and machine noise. Both infant cries reduced the mothers’ concentration levels more than the machine noise. In a study by Chang and Thompson [23], adults were asked to calculate simple subtraction problems while listening to attachment vocalizations (whines, cries, etc.) as well as to control sounds; participants were more distracted when listening to attachment vocalizations than to control sounds, regardless of gender or parental status. More recently, Hechler, Beijers, & de Weerth [24] showed that participants made the most mistakes on a working memory task when listening to infant crying, compared to other disturbing noises. Although much work has shown an attention bias to infant vocal stimuli, in which the power of infant vocalizations to grab attention subsequently interferes with information processing, the more specific types of neurocognitive processes affected by this attention bias to infants have remained elusive.

### Current study

In the current study, we sought to characterize the cognitive and cortical demands of infant vocal distractors (cries vs. laughs) on attention during a conflict task using an electroencephalography (EEG) and event-related potential (ERP) design.

In conflict tasks, performance is typically slower and the neural responses associated with cognitive control and conflict processing are greater during incongruent than during congruent trials [25–28]. In the classic Stroop task [29], larger amplitudes in the N400 are seen over the frontocentral regions for incongruent trials compared to congruent or neutral trials, suggesting an increased allocation of attention as well as enhanced cognitive conflict for the incongruent condition [26–28]. Neural generators of the N400 have been localized to the PFC and ACC [28].

Less established are the effects of emotional distractors (such as negative and/or positive emotional cues) on electrocortical response to cognitive conflict. In terms of the effects of emotional distraction on conflict processing, it has been found that there is greater amplitude in the N450 and slower performance in response to negative vs. positive emotional distractors during a conflict task [30–32]. In terms of the effects of emotional distraction on earlier attention processing, it has been found that emotional compared to neutral stimuli [33, 34] or happy compared to sad stimuli [35] enhance attention, as indexed by greater amplitude in the P200 ERP [30, 31, 36]. Greater positive peak in the amplitude of the P200 component in response to emotional targets is detected over centroparietal regions, with evidence for greater right hemispheric activity [37].

Because previous studies did not consider pleasant infant vocalizations, we asked whether infant crying had particular force vs whether a positive vocalization (e.g., infant laughing) might also modulate attention. Furthermore, previous studies on the distracting power of infant cries employed paradigms in which infant vocalizations were presented during a cognitive task; by contrast, we sought to explore whether sequential (Experiment 1) vs. simultaneous (Experiment 2) presentation of infant vocal distractions would differentially affect performance. Specifically, we evaluated the effects of infant vocal distractors on early (P200 ERP) and late cortical responses (N450 ERP) associated with attention and conflict processing during a Stroop task [29]. By considering the effects of infant distraction on a cognitive conflict task, we examined whether the valence of infant vocalizations (cries vs. laughs) differentially modulates attention.

We hypothesized that as a result of the negative arousal bias [38, 39] infant cry vocalizations would evoke greater distraction than infant laugh vocalizations during a cognitive conflict task. Specifically, we hypothesized, participants would show increased difficulty shifting attention away from the infant cry vocalization to the Stroop task, thereby interfering with task performance, regardless of whether infant vocalizations were presented before (Experiment 1) or during Stroop trials (Experiment 2). Furthermore, we expected Stroop trials paired with infant cry vocalizations to diminish attention, as indexed by smaller P200 ERP responses to Stroop trials, and elicit greater conflict processing, as indexed by larger N450 ERP responses to Stroop trials, compared with infant laugh vocalizations.

## Experiment 1 (Sequential Presentation): Methods

### Participants

15 right-handed, nulliparous, unmarried students from the University of Toronto (7 females; *M*_age_ = 20.9 years, *SD* = 4.7) participated for course credit, signed an informed consent agreement, and were debriefed after the study. Our sample size for Experiment 1 was chosen because previous studies examining the valence of non-infant distractors during conflict tasks found valence effects on cortical processes with similar sample sizes [40, 41]. For example, Lv et al. (2011) demonstrated a valence effect on the P3 ERP component (*mean difference* = 3.5 microvolts) during an auditory-visual distraction paradigm with a similar sample size (*N* = 12). None of the participants reported suffering from medical or psychiatric conditions or problems that could affect hearing or vision, and none reported taking any prescription medication in the past two weeks. EEG data from two participants were missing due to technical errors. Only participants with complete EEG and behavioral data were included in the analyses. In addition, one subject was removed due to extreme values in ERP data. Thus, results presented are based on *N* = 12 participants (6 females). The Ethics Review Office at the University of Toronto approved all procedures.

### Infant stimuli

**Fig 1:**
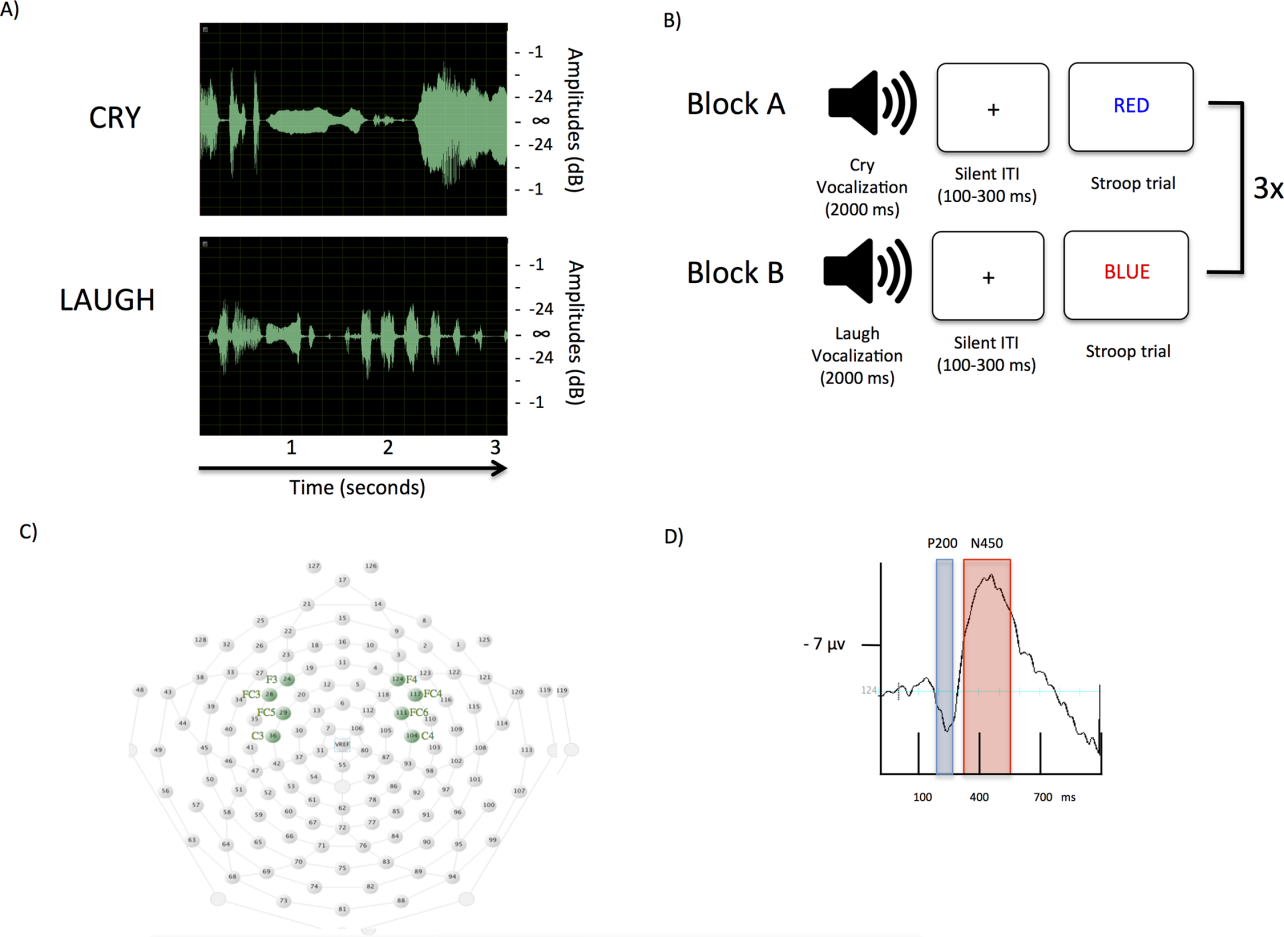
Experimental methods. (A) Amplitudes (dB) of the infant cry and laugh vocalizations. (B) Schematic of the cross-modal distraction paradigm: Block A and B each consist of 24 trials and are repeated three times in an A-B-A-B-A-B design for a total of 144 trials. For each trial, an infant vocalization is presented for 2000 miliseconds (ms) followed by a Stroop trial. The interstimulus interval (ISI) between the vocalization and Stroop trial was 100–300 ms and consisted of a fixation cross “+” presented in silence. (C) Map of recording electrodes. Electrocortical responses to Stroop trials were analyzed from 8 electrodes (denoted by the green circles), which included the left frontal electrode # 24 (F3), the left frontal-central electrodes # 28 and # 29 (FC3 and FC5), the left central electrode # 36 (C3), the right frontal electrode # 124 (F4), the right frontal-central electrodes # 111 and # 117 (FC4, FC6), and the right central electrode # 104 (C4). (D) The grand average waveform depicting the temporal window for the P200 (128–260 ms) and N450 (350–550 ms) ERPs at the right frontal electrode #124 (F4).

Standardized recordings of infant vocalizations were obtained from the International Affective Digitized Sounds system (IADS)[42]. Two infant sounds were used, crying and laughing (sample #261 cry and #110 laugh); these were presented via speakers and matched for duration and volume (see Fig 1A). The sound pressure level was maintained at a peak amplitude of -3.67 dB and set to a comfortable listening volume by the experimenter; this volume remained stable for all participants. Participants completed affect ratings of the infant stimuli using the 9-point Self-Assessment Manikin scale (SAM)[43]. The two primary dimensions analyzed were affective valence (ranging from pleasant to unpleasant) and arousal (ranging from calm to excited). Participants rated the infant cry as more arousing (*M* = 6.62, *SD* = 0.87) relative to the infant laugh (*M* = 4.23, *SD* = 1.1), *t* (12) = 6.82, *p* < 0.001. In addition, participants rated the infant laugh as more pleasant (*M* = 7.46, *SD* = 1.2) relative to the cry (*M* = 4.38, *SD* = 1.4), *t* (12) = -5.0, *p* < 0.001. Participants also rated the extent of their daily experience with infants on a Likert scale ranging from 1 *(none)* to 5 *(a lot)*, *M* = 2.92, *SD* = 1.16 and rated their general feeling toward infants on a Likert scale ranging from 1 *(extremely negative)* to 5 *(extremely positive)*, *M* = 3.92, *SD* = 0.79.

### Stimuli and procedure

Fig 1B depicts a diagram of the design of Experiment 1. Visual stimuli were shown on a 48.3-cm computer screen (70 Hz refresh rate) that was located 40–50 cm from the viewer. The experiment consisted of one practice block of 36 Stroop trials, followed by six experimental blocks each consisting of 24 sequential infant distraction Stroop trials (total of 144 trials). Three infant cry blocks (Block A) and three infant laugh blocks (Block B) were used. To minimize habituation, the order of laugh and cry blocks was alternated in an A-B-A-B-A-B design. In all trials, an audio recording of the infant vocalization was presented on headphones for 2000 ms followed by the presentation of a visual Stroop trial (2000 ms).

For each trial, the infant vocalization was played for 2000 ms and paired with a fixation cross (+), which was presented at the center of a computer monitor (in black with a white background). The fixation cross was used to minimize eye movements and EEG artifact. Between the vocalization and start of a Stroop trail, the fixation cross was randomly presented for 100–300 ms to serve as a silent interstimulus interval (ISI). The total time between Stroop trials ranged from 2100 to 2300 ms. Each Stroop trial had a fixed duration of 2000 ms and consisted of colored words that were presented in the center of the screen against a white background. Each word was shown in one of four colors (red, blue, green, and yellow). Color words randomly appeared as either congruent (e.g., the word “RED” printed in red; mean number of trials = 48.83), incongruent (e.g., the word “RED” printed in blue; mean number of trials = 47.92), or control trials (e.g., the letters “XXX” in red; mean number of trials = 47.25). Participants were instructed to ignore the audio playback/soundtrack and to indicate the color of a target word (red = 1, blue = 2, green = 3, and yellow = 4) by pressing a key on a computer keyboard (the keys corresponded to the four fingers of their preferred hand). Reaction times (RT) and number of errors were recorded.

### EEG recording

EEG was recorded using a 128-electrode Hydrocel Geodesic Sensor Net (EGI, Eugene, OR). EEG was sampled at 250 Hz, with band-pass filters set at 0.1–100 Hz, referenced to the vertex, recorded with 20K amplification, with a 40 Ω impedance. Recordings were digitally filtered with a 40-Hz low-pass filter and were re-referenced to the average reference prior to analysis. The EEG sensor net was fitted so that the electrodes were spaced evenly and symmetrically to cover the scalp from nasion to inion and from left to right ear.

Following recording, EEG was segmented into 1100-ms segments beginning 100 ms before target onset and ending 1000 ms after target presentation. Baseline correction was performed using the 100 ms prestimulus interval. Preprocessing of the trials was conducted using the Net Station (4.0) artifact detection tool. Segments in which the signal exceeded 150 μV were identified as bad channels, 140 μV as eye blinks, and 40 μV as eye movements. Channels with artifacts in more than 20% of trials were marked as bad, and data from bad channels were replaced with data interpolated from the good channels. Segments with more than 10 bad channels were excluded from the analysis. A mean of 123 artifact-free epochs (85.42% of total trials) was calculated separately for each electrode, condition, and individual. Fig 1C presents a map of the 8 recording electrodes that were analyzed. These locations were selected in previous studies examining the effects of emotional distraction on attention and conflict processing [30, 36].

### ERP scoring

To examine the effects of valence of infant vocalization on attention and conflict monitoring, we visually identified temporal components for the P200 (128–260 ms) and N450 (350–550 ms) ERP components and scored their peak amplitude activity. Fig 1D shows a grand average waveform highlighting the temporal window for the P200 and N450 ERP components. These two ERPs were time-locked to target onset of Stroop trials.

### Data analysis

Two separate tests were conducted on reaction times (RT) and number of errors using a 2 x 3 repeated-measures ANOVA with infant vocalizations (cry vs. laugh) and Stroop type (congruent, incongruent, and control) as the within-subject factors. Amplitudes of the P200 and N450 components were analyzed separately using a 2 x 3 x 4 x 2 ANOVA with the repeated factors of valence (infant cries and laughs), Stroop (congruent, incongruent, and control trials), location (frontal, twice frontal-central, central) and hemisphere (right, left). Statistical analyses were conducted on ERP data that was winsorized to restore a normal distribution. To determine the associations between attention and conflict processing, Pearson correlations were conducted on the peak amplitudes of the P200 and N450 components for correct trials primed with cries vs. laughs. Multiple regression analysis was conducted to determine the associations among ERPs and RT. For all analyses, effect sizes were calculated using the partial eta-squared function and significance was set at *p <* 0.05. A Bonferroni correction for multiple comparisons was applied. Incorrect Stroop trials (2.60% of total trials) were removed from analyses of RT and ERP data.

## Experiment 1 (Sequential Presentation): Results

The data from Experiment 1 is available in the supporting information (S1 Table). Preliminary analyses revealed that age (*p* = 0.11), sex *(p* = 0.42), participants’ ratings of pleasure (*p* = 0.87) and arousal (*p* = 0.25) demonstrated by the laughs and cries, extent of infant experience (*p* = 0.75), and reported feeling toward infants (*p* = 0.19) did not have significant effects on Stroop performance. These variables were therefore excluded in the main analysis. A Shapiro-Wilk test of normality was non-significant for cry primed trials, *W* (12) = .94, *p* = 0.48, and laugh primed trials, *W* (12) = .95 *p* = 0.63, providing no evidence that data deviated from normality. Visual inspection of histograms, normal Q-Q plots, and box plots revealed cry and laugh primed Stroop trials were normally distributed, with a skewness of -0.35 (*SE* = 0.64) and -0.17 (*SE* = 0.64) respectively, and kurtosis of -0.95 (*SE* = 1.23) and -1.09 (*SE* = 1.23) respectively.

### Behavioral data

**Fig 2:**
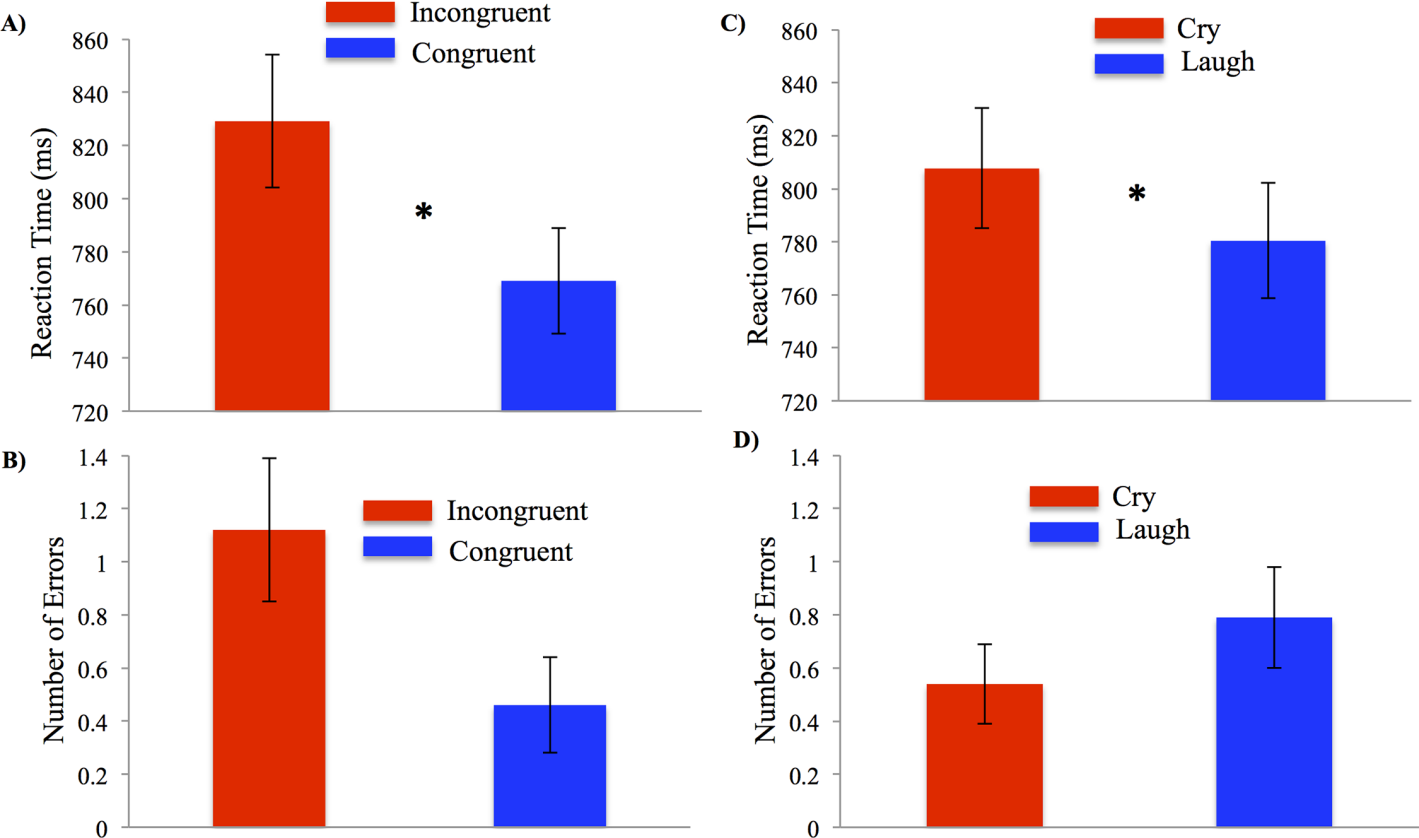
Performance on the sequential cross-modal distraction paradigm differed by Stroop condition and valence of infant vocalizations in Experiment 1. (A) Mean reaction time (in milliseconds, ms) and number of errors (B) for incongruent (red bars) and congruent trials (blue bars). Mean reaction time (C) and number of errors (D) for cry (red bars) and laugh (blue bars) conditions. Asterisks (*) represent statistical significance at *p* < .01 value. Error bars represent standard errors.

RT were shorter for congruent (*M* = 769.05 ms, *SE* = 19.86; *p* = 0.002) and control (*M* = 783.93 ms; *SE* = 23.57; *p* = 0.007) trials compared to the incongruent trials (*M* = 829.16 ms, *SE* = 25.05) demonstrating the standard Stroop effect, *F* (2, 22) = 14.33, *p* < 0.001, η^2^ = 0.57 (see Fig 2A). There was also a significant main effect of infant valence, *F* (1, 11) = 12.29, *p* = 0.005, η^2^ = 0.53, in which participants were slower on trials cued with infant cries (*M* = 807.72 ms; *SE* = 22.70) than trials cued with infant laughs (*M* = 780.38 ms; *SE* = 21.77; see Fig 2C). There were no main effects of Stroop type on number of errors (*p* = 0.09; see Fig 2B) or of valence on number of errors (*p* = 0.11; see Fig 2D) and no significant Stroop type by valence interactions (*p* = 0.18).

### EEG/ERP data

#### N450 ERP

A Shapiro-Wilk test and visual inspection of histograms, normal Q-Q plots and box plots revealed that N450 amplitudes for all cry trials were normally distributed, *W* (12) = 0.91, *p* = 0.20, with a skewness of -0.26 (*SE* = 0.63) and kurtosis of -1.55 (*SE* = 1.23). Data for all laugh trials was also normally distributed, *W* (12) = 0.88, *p* = 0.09, with a skewness of -0.28 (*SE* = 0.64) and kurtosis of -1.72 (*SE* = 1.23).

**Fig 3:**
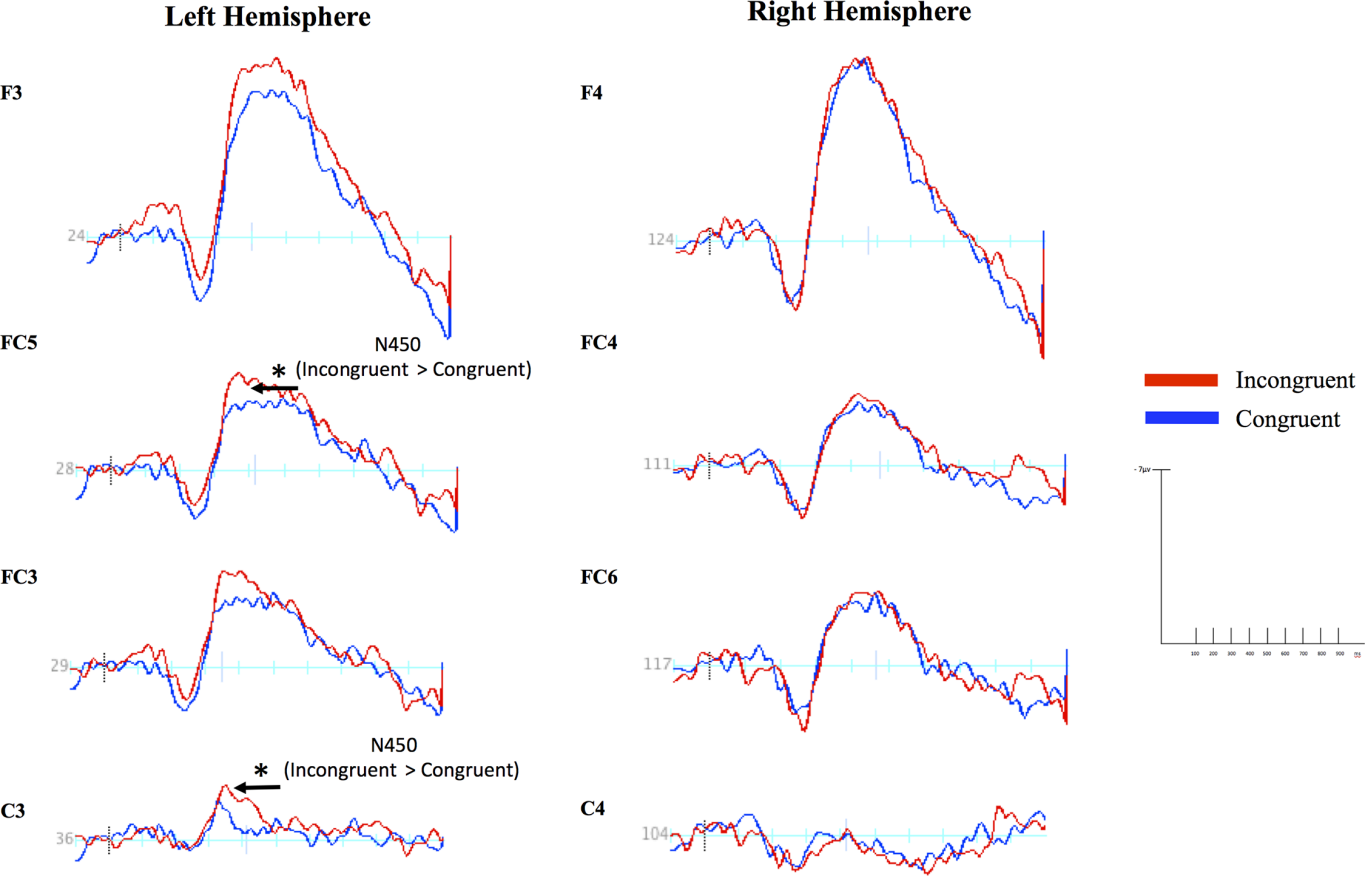
Electrocortical responses differed by Stroop condition in the left hemisphere. Average event-related brain potential (ERP) waveforms (in microvolts) for incongruent (red lines) and congruent trials (blue lines) at all locations. Asterisks (*) denote statistical differences between incongruent and congruent trials for the N450 ERP with a significance level at *p <* .01, while arrows with (*t*) indicate a trend at *p* < 0.10.

Results of the ANOVA revealed a main effect of Stroop, *F* (2, 22) = 4.40, *p* = 0.025, η^2^ = 0.28. Paired t-tests conducted as post hoc revealed larger amplitudes for incongruent (*M* = -6.41, *SE* = 0.94) than congruent trials (*M* = -5.63, *SE* = 0.92), *t* (11) = -2.12, *p* = 0.05, and larger amplitudes for control (*M* = -6.44, *SE* = 1.0) than congruent trials, *t* (11) = 2.40, *p* = 0.03. These findings are consistent with literature on the Stroop effect and N450 ERP [26–28]. Additional t-tests conducted as follow-up further showed that the Stroop effect was lateralized to the left hemisphere, with larger negativity for incongruent compared to congruent trials at left central site C3, *t* (11) = -3.43, *p* = 0.006, and slightly larger at left frontal-central location FC5, *t* (11) = -2.08, *p* = 0.06, with no differences found for sites FC3, *t* (11) = -1.37, *p* = 0.19 or F3, *t* (11) = -1.06, *p* = 0.31. The Stroop effect was not evident at any electrodes in the right hemisphere (F4, *t* (11) = -1.53, *p* = 0.16; FC4, *t* (11) = -0.66, *p* = 0.52; FC6, *t* (11) = -1.48, *p* = 0.17; C4, *t* (11) = -0.63, *p* = 0.54). Fig 3 shows average ERP waveforms for incongruent and congruent trials at all locations.

Our ANOVA model further revealed a main effect of location, *F* (3, 33) = 26.09, *p* < 0.001, η^2^ = 0.70, with larger N450 amplitudes for frontal sites F3 and F4 (*M* = -10.59, *SE* = 1.8) than central sites C3 and C4 (*M* = -2.14, *SE* = 0.47; *p* = 0.001), frontal-central sites FC3 and FC4 (*M* = -5.61, *SE* = 0.78; *p* = 0.005) and FC5 and FC6 (*M* = -6.24, *SE* = 0.93; *p* = 0.01). Greater N450 amplitudes were also found for FC4 and FC5 as well as FC5 and FC6 than C3 and C4 (*p* < 0.001; *p* < 0.001). More interestingly, results showed a valence x Stroop x location x hemisphere interaction, *F* (6, 66) = 3.81, *p* = 0.003, η^2^ = 0.26.

**Fig 4:**
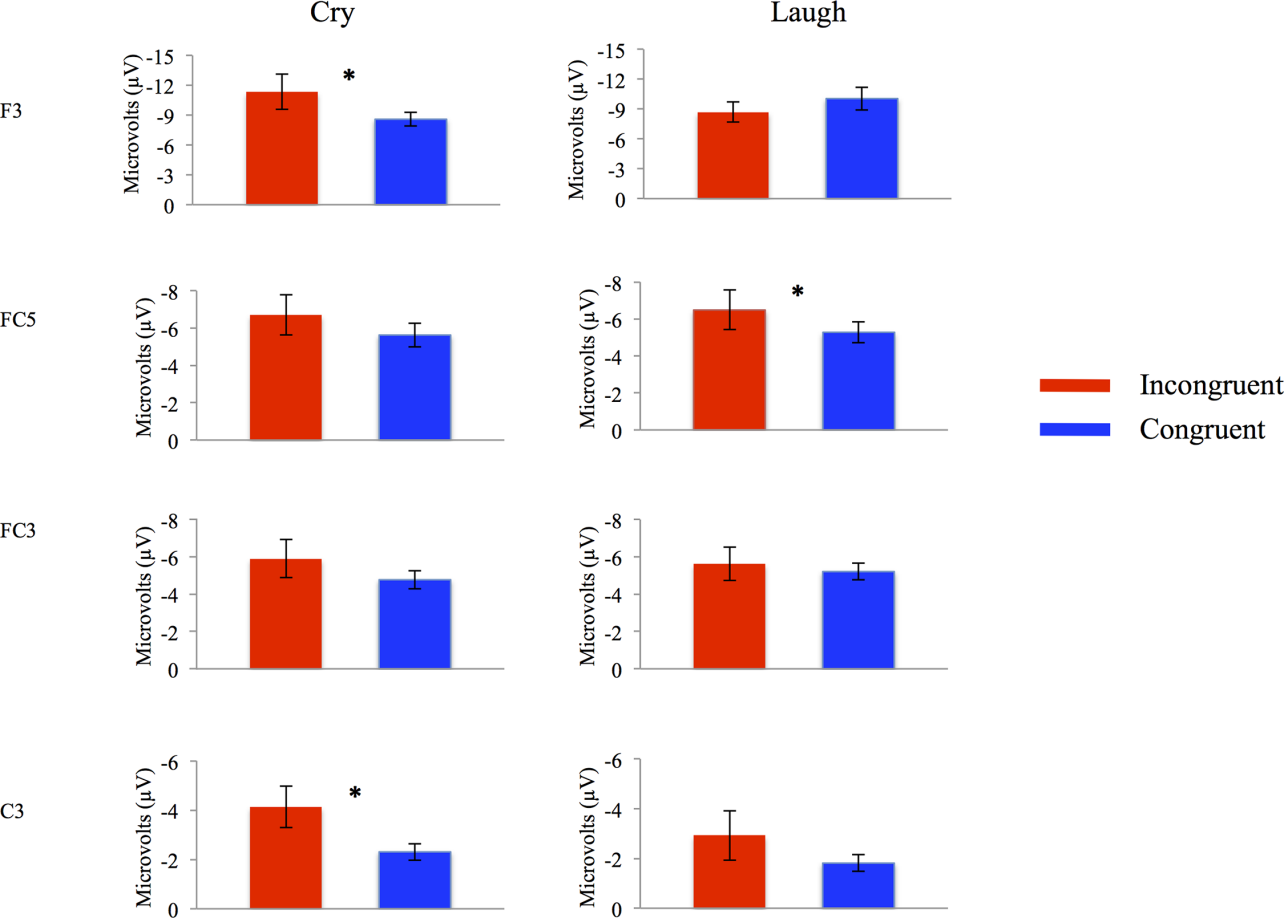
The Stroop effect on the N450 ERP occurred in the frontal and central locations after infant cries and in the frontal-central location after infant laughs. Mean amplitudes (in microvolts) of the N450 ERP by incongruent (red bars) and congruent trials (blue bars) for electrodes in the left frontal (F3), the left frontal-central (FC5/FC3), and the left central locations (C3). Cry and laugh blocks are presented in the left and right columns, respectively. The asterisks (*) denote statistical significance values at *p* < .01. Error bars represent the standard errors around the mean.

To break down this interaction, difference scores were generated between Stroop trials primed with infant cry and laugh vocalizations for each location and hemisphere. The effects of Stroop trial on location and hemisphere were tested using Student’s t-tests. The Stroop effect (larger amplitudes for incongruent than congruent trials) was found for trials primed with infant cries in the left hemisphere at frontal site F3, *t* (11) = -3.47, *p* = 0.005, and central site C3, *t* (11) = -3.05, *p* = 0.01 (see Fig 4). These effects were localized to the left hemisphere, and were not present for frontal site F4, *t* (11) = 0.12, *p* = 0.91, or central site C4, *t* (11) = -1.39, *p* = 0.19 in the right hemisphere (see Fig 3).

The Stroop effect was additionally significant for trials primed with infant laughs in the left hemisphere at frontal-central site FC5, *t* (11) = -2.71, *p* = 0.02 (see Fig 4), but not in the right hemisphere at frontal-central site FC6, *t* (11) = -1.36, *p* = 0.19.

**Fig 5:**
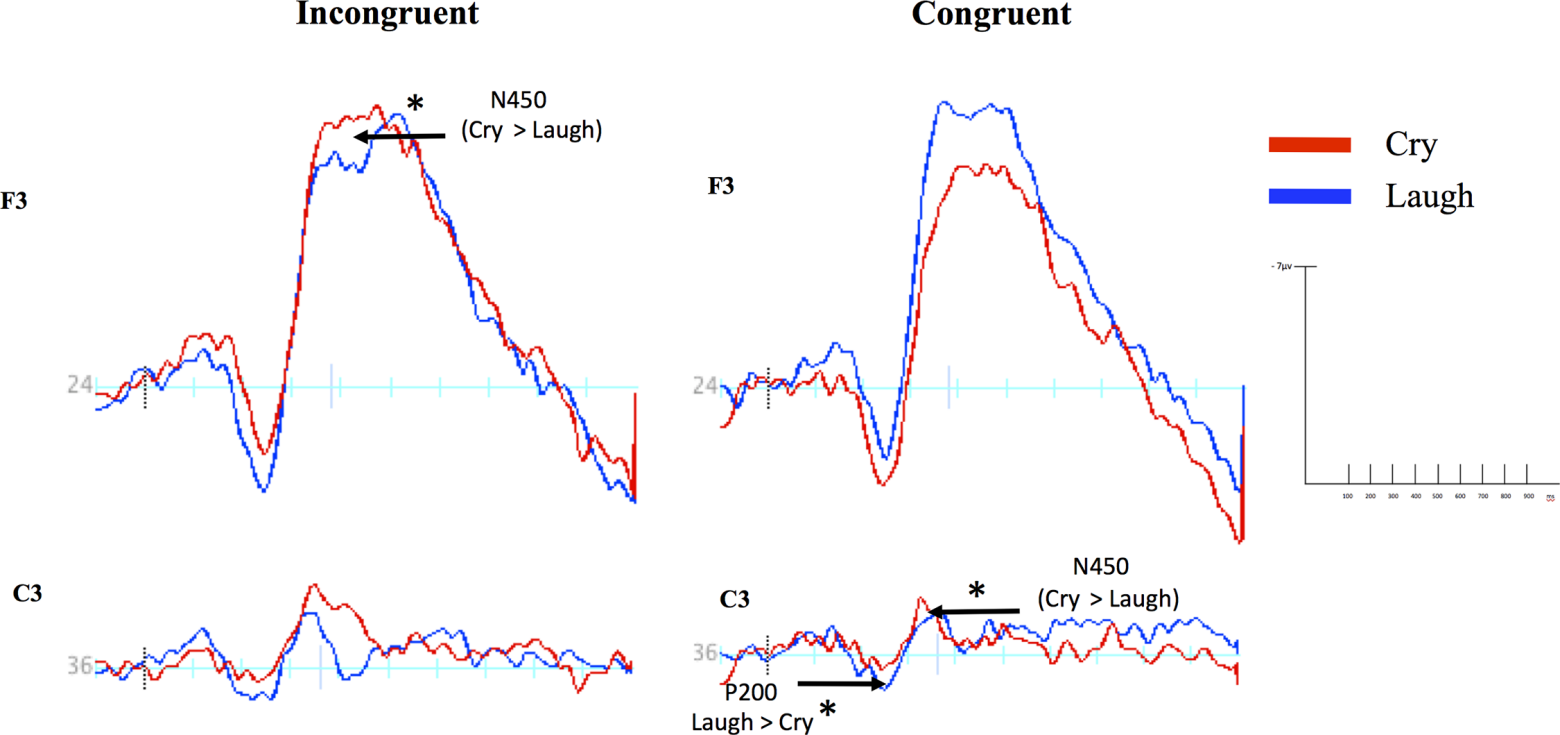
The cry effect on electrocortical responses during incongruent and congruent trials occurred in the frontal and central locations, respectively. Waveforms presented (in microvolts) cry (red lines) and laugh blocks (blue lines) at left frontal (F3) and left central (C3) locations for both incongruent (left column) and congruent trials (right column). Asterisks (*) denote statistical differences between cry and laugh ERPs with a significance level at *p <* .01.

Given that the above Stroop effects were found to be localized in the left hemisphere, potential valence effects on location in the left hemisphere were also tested using Student’s t-tests (see Fig 5). Results revealed larger amplitudes for incongruent Stroop trials primed with cries than laughs at frontal site F3, *t* (11) = -2.24, *p* = 0.04, and larger amplitudes for congruent trials primed with cries than laughs at central site C3, *t* (11) = -2.34, *p* = 0.04 (See Fig 5).

#### P200 ERP

A Shapiro-Wilk test and visual inspection of histograms, normal Q-Q plots and box plots showed that P200 amplitudes for all cry trials were normally distributed, *W* (12) = 0.95, *p* = 0.63, with a skewness of -0.55 (*SE* = 0.63) and kurtosis of 0.27 (*SE* = 1.23). Data for all laugh trials was also normally distributed, *W* (12) = 0.94, *p* = 0.46, with a skewness of 0.24 (*SE* = 0.64) and kurtosis of 0.004 (*SE* = 1.23).

Results from the ANOVA revealed a main effect of location, *F* (3, 36) = 8.74, *p* < 0.001, η^2^ = 0.44, with larger amplitudes in frontal sites F3 and F4 (*M* = 3.98, *SE* = 0.43) than frontal-central sites FC3 and FC4 (*M* = 2.98, *SE* = 0.24; *p* = 0.03) and slightly larger amplitudes than central sites C3 and C4 (*M* = 2.61, *SE* = 0.19; *p* = 0.06). Larger amplitudes were also seen in frontal-central sites FC5 and FC6 (*M* = 3.49, *SE* = 0.24) than C3 and C4 (*p* = 0.01) and FC3 and FC4 (*p* = 0.01). Results further revealed a Stroop x hemisphere interaction, *F* (2, 22) = 3.54, *p* = 0.04, η^2^ = 0.24, and a valence x location x hemisphere interaction, *F* (3, 33) = 3.64, *p* = 0.02, η^2^ = 0.25. To break down these interactions, difference scores were generated between cry and laugh primed Stroop trials for each location and hemisphere. The effects of valence on location and hemisphere were then tested using Student’s t-tests. Results revealed smaller amplitudes for congruent Stroop trials primed with infant cries than infant laughs in the left hemisphere at central site C3, *t* (11) = -2.41, *p* = 0.03 (see Fig 5), but not at central site C4 in the right hemisphere, *t* (11) = -0.15, *p* = 0.88. Results further showed slightly smaller amplitudes for cry than laugh congruent Stroop trials in the right hemisphere at frontal-central locations FC4, *t* (11) = -1.78, *p* = 0.1, and FC6, *t* (11) = -2.03, *p* = 0.06, but not in the left hemisphere at frontal-central sites FC3, *t* (11) = 0.30, *p* = 0.76 and FC5, *t* (11) = -1.12, *p* = 0.28. Amplitudes for incongruent Stroop trials primed with infant cries were also slightly smaller than trials primed with infant laughs in the left hemisphere at central site C3, *t* (11) = -1.96, *p* = 0.07, but not in the right hemisphere at central site C4, *t* (11) = -0.29, *p* = 0.77.

### Correlations between P200 and N450

The peak amplitudes of the P200 and N450 components were correlated to each other in trials primed with infant cries (*r* = -0.66, *p* = 0.02) and in trials primed with infant laughs (*r* = -0.64, *p* = 0.02).

Experiment 1 revealed that infant cries were more distracting than laughs. We asked whether this valence effect on attention would be similar when infant distractors were presented simultaneously with the cognitive task.

## Experiment 2 (Simultaneous Presentation): Methods

### Participants

20 right-handed nulliparous, unmarried, female University of Toronto students (*M*_age_ = 20.6 years, *SD* = 2.7 years) participated, signed an informed consent, and were debriefed after the study. Our sample size in Experiment 2 was selected based on prior work showing valence effects of emotional distraction on reaction time during conflict tasks [40, 41, 44]. None of the participants reported suffering from a medical or psychiatric condition, a problem that could affect hearing or vision, and none noted taking any prescription medication in the past two weeks. Behavior data from two participants were incomplete and not included in the analyses; results presented are based on *N* = 18 participants. Incorrect Stroop trials (4.01% of trials) were removed from analyses of RT data. The Ethics Review Office at the University of Toronto approved all procedures.

### Procedure and stimuli

Experiment 1 and 2 used the same procedure and stimuli except for the timing of the emotional distractors, where in Experiment 2 the distractors were presented simultaneously with the Stroop trials.

## Experiment 2 (Simultaneous Presentation): Results

The data from Experiment 2 is available in the supporting information (S2 Table). A Shapiro-Wilk test of normality was non-significant for cry primed trials, *W* (18) = .94, *p* = 0.28, and for laugh primed trials, *W* (18) = .94 *p* = 0.98, providing no evidence that data deviated from normality. Visual inspection of histograms, normal Q-Q plots, and box plots revealed cry and laugh primed Stroop trials were normally distributed, with a skewness of -0.55 (*SE* = 0.54) and -0.77 (*SE* = 1.03) respectively, and kurtosis of 0.32 (*SE* = 0.54) and -0.38 (*SE* = 1.03) respectively.

**Fig 6:**
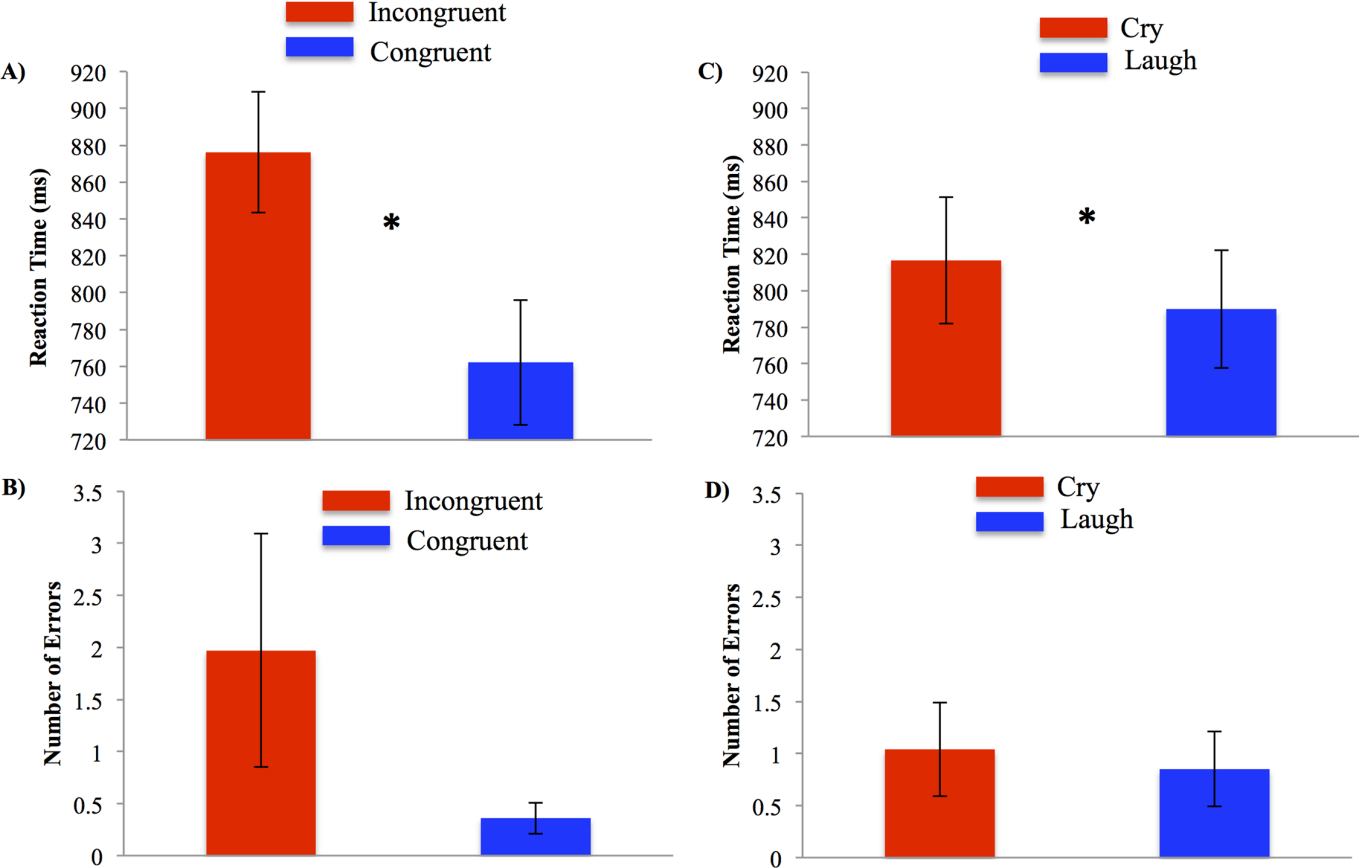
Performance on the simultaneous cross-modal distraction paradigm differed by Stroop condition and valence of infant vocalizations in Experiment 2. (A) Mean reaction time (in milliseconds, ms) and number of errors (B) for incongruent (red bars) and congruent trials (blue bars). Mean reaction time (C) and number of errors (D) for cry (red bars) and laugh (blue bars) conditions. Asterisks (*) represent statistical significance at *p* < .01 value. Error bars represent standard errors.

Participants were significantly slower for incongruent trials (*M* = 876.14 ms, *SE* = 32.70) than for congruent trials (*M* = 762.11 ms, *SE* = 33.97) and control trials (*M* = 771.64, ms *SE* = 35.81), *F* (2, 34) = 41.32, *p* < 0.001, η^2^ = 0.71, see Fig 6A. Consistent with the results of Experiment 1, the results of Experiment 2 revealed a main effect of valence on RT, *F* (1, 17) = 6.92, *p* = .02, η^2^ = 0.29, see Fig 6C. Participants performed the task more slowly when simultaneously presented with an infant cry (*M* = 816.64 ms, *SE* = 34.66) than with an infant laugh (*M* = 789.92 ms, *SE* = 32.31). There was no valence and Stroop type interaction, *F* (2, 34) = 1.65, *p* = .20, η^2^ = 0.09. Valence and Stroop type did not affect the number of errors made, *F* (1, 17) = 2.83, *p* = .11, η^2^ = 0.14; *F* (2, 34) = 1.87, *p* = .17, η^2^ = .09, respectively, see Fig 6B and 6D.

To compare the behavior results from Experiment 1 and Experiment 2, a valence x Stroop multivariate analysis of variance (MANOVA) with Experiment as a fixed factor conducted separately for RT and accuracy showed that the pattern of results from Experiment 1 is the same as the results from Experiment 2 for RT, *F* (6, 23) = 1.99, *p* = 0.11, η^2^ = 0.34, and for accuracy, *F* (6, 23) = 0.96, *p* = 0.47, η^2^ = 0.20. Thus, the effects of emotional distraction produced by infant vocalizations on cognitive performance are similar when infant distractors are presented sequentially or simultaneously with a cognitive task.

## Discussion

Our finding that infant cries disrupt attention and increase conflict processing underscores the unique impact of infant vocalizations on cognitive control. To characterize the cognitive and cortical demands of infant cries, we evaluated the effects of infant cry and laugh vocalizations on attention and conflict processing, as indexed by the P200 and the N450 ERPs, respectively, during a Stroop task. Although the infant cries disrupted behavioral performance equally on congruent and incongruent trials, they modulated neurocognitive processes differently during congruent and incongruent trials. For incongruent trials, infant cries elicited greater conflict processing (larger N450) in the left frontal cortex compared to laughs. For congruent trials, infant cries diminished attention (smaller P200) and elicited greater conflict processing (larger N450) in the left central cortex compared to laughs. Thus, for more demanding trials, cries elicited greater conflict processing in frontal regions, and for less demanding trials, cries diminished attention and elicited greater conflict processing in central regions. The findings are consistent with the existence of a negative arousal bias to infant cries, which selectively and differentially modulates neurocognitive processes in the frontal and central locations of the left hemisphere depending on the cognitive demands of the task.

Our behavior findings are consistent with prior work on the interfering effects of infant cries on attention and working memory [22–24]. It should be noted that the magnitude of the cry interference effect on Stroop performance was relatively small (mean _cry–laugh blocks_ = 28 ms) compared to the magnitude of the Stroop interference effect (mean _incongruent–congruent trials_ = 98 ms). Nevertheless, the cry interference effect was replicated in two experiments, in which vocalizations were presented before (Experiment 1) or during Stroop trials (Experiment 2). It should also be noted that infant vocalizations did not affect rates of accuracy in either experiment, suggesting that cry interference effect was not due to task errors.

Several findings and models from the cognitive neuroscience literature are relevant to discussing the effects of emotional distraction on conflict processing in this context. First, previous work has shown that negative emotions influence cognitive control through a negative bias, resulting in prioritized attention to emotional stimuli [38, 39, 45, 46]. More specifically, Xue et al. [32] showed larger N450 amplitudes during incongruent than congruent trials for emotional vs. non-emotional stimuli indicating that greater conflict processing is generated by the combination of negative arousal and cognitive demand. The neural generators of the N400/N450 have been localized to the PFC and ACC [25, 28]. Imaging research has further shown that the N450 is a valid index of cognitive conflict and suggests that the N450 generator is localized in the ACC, which is a crucial modulator of both cognitive and emotional conflict [32, 47–49]. Although areas of the ACC involved in emotional and cognitive processing are anatomically distinct, Drevets and Raichle [50] reported a reciprocal suppression pattern of blood flow, in which blood flow to areas that serve cognitive functions was reduced during negative emotional states. In turn, functional connectivity between the ventral and dorsal anterior cingulate (ACC) is increased during incongruent trials paired with an emotional versus neutral stimulus, suggesting that the ACC is responding to the increased need for cognitive control in response to a conflict challenge paired with a distracting and/or distressing emotional stimulus. These findings support the possibility that reciprocal suppression occurring in subregions of the ACC also play a role in how infant vocalization modulates cognition and diminishes cognitive performance.

Whether the cry interference effect is initiated by attention depletion followed by enhanced conflict processing or is driven by enhanced conflict processing, which then inhibits attention is open to speculation. Previous work suggests that coping with task-irrelevant emotional distraction recruits activity and enhances interactions between brain regions involved in emotion processing (e.g., the amygdalae, ventrolateral PFC (vlPFC), and the medial PFC) and cognitive control (e.g., dorsolateral prefrontal cortex (dlPFC) and the lateral parietal cortex (LPC) [51–53]. Engagement of the lateral and medial PFC further enhances functional coupling with activity in the amygdala [51]. Given the demands that incongruent trials place on executive and working memory processes, our findings show that cries may diminish activity in prefrontal areas, thereby hindering performance, potentially due to their heavy demand on activating regions associated with basic emotional processing. Our findings thus suggest that the sequence of neural activity underlying these two basic cognitive processes (attention and conflict processing) may differ depending on the cognitive demands of the task.

The experiments reported here provide new insight into the cognitive demands elicited by infant cries and laughs. However, several limitations are worth noting: First, small sample sizes were used in both experiments. Although it is heartening that the results of Experiment 1 were replicated in Experiment 2 using a modified version of the design, and although this suggests that the sample size is sufficient, it will be important to replicate both the behavioral and electrocortical findings in a study with a larger sample size. Second, the inclusion of only two types of infant vocalizations (cries and laughs) means that this study is unable to address the question of how or whether the effects of infant vocalizations on cognition differ from the effects of other noises or sounds. Third, we used task-irrelevant distractors; task-relevant distractors could be used in future experiments to determine whether the cry interference effect could be reversed [53]. Fourth, our analysis of electrocortical data limits discussion of brain localization. Future research using a dual fMRI-EEG paradigm to further investigate the sources and timing of neural responses to the cry interference paradigm would be worthwhile. Fifth, the experiments evaluated non-parents and therefore do not address whether parental status and experience affect infant salience and effects on cognition. Previous ERP research examining the effects of parental status on the orienting response to infant cries found larger N100 responses in mothers than in non-mothers [18, 54]. Whether this potentially enhanced alertness to the infant cry in mothers would act to more severely disrupt attentional processing on a conflict task would be of interest.

## Conclusion

Overall, our findings highlight a paradox in evolutionary programming that may apply to both parents and potential parents. In addition to confirming the long-recognized power of the infant cry to capture attention, our study shows that the infant cry challenges the adult brain’s capacity to engage in parallel processing—to simultaneously distinguish between and process two distinct streams of information (apprehending color while reading verbal cues). While the standard evolutionary view posits that the infant cry’s power to capture our attention is adaptive, this view does not address the potential costs of attention depletion in the caregiver. Nor does it address the possible practical benefits of a caregiver’s momentarily ignoring the infant cry in order to shift and attend flexibly to multiple demands—a skill that presumably would benefit both the quality of infant care and the well-being of caregivers. An alternative view suggested by our findings emphasizes not only the evolutionarily adaptive function of the caregiver’s automatic stimulus response to the infant cry—a response that has basic survival value—but also the potentially adaptive function of the cognitive conflict that the infant cry elicits, which may foster a cognitive flexibility that enables the caregiver to selectively respond both to infant vocal distress and to competing demands, rapidly switching between them and thereby optimizing environmental resources.

## Supporting Information

S1 Table Data set from Experiment 1. (XLSX)

S2 Table Data set from Experiment 2. (XLSX)
